# ‘*It’s almost superstition: If I don’t think about it, it won’t happen*’. Public knowledge and attitudes towards advance care planning: A sequential mixed methods study

**DOI:** 10.1177/02692163211015838

**Published:** 2021-05-17

**Authors:** Sonja McIlfatrick, Paul Slater, Olufikayo Bamidele, Deborah Muldrew, Esther Beck, Felicity Hasson

**Affiliations:** 1 School of Nursing, Institute of Nursing and Health Research, Ulster University, Newtownabbey, Northern Ireland; 2 Institute of Clinical and Applied Health Research, Hull York Medical School, University of Hull, Hull, UK

**Keywords:** Advance care planning, mixed methods, knowledge, attitudes, public

## Abstract

**Background:**

Internationally, participation in advance care planning is low. Whilst a community action approach is advocated, what the public know and understand about advance care planning is unknown.

**Aim:**

To assess public awareness, knowledge and attitudes towards advance care planning and identify strategies to raise awareness within a public health framework.

**Design:**

Sequential mixed methods comprising a cross-sectional survey and focus group/interviews.

**Setting/participants:**

A random representative sample of adults from one region of the United Kingdom (*n* = 1201; response rate 56%) completed a face-to-face survey. Twenty-five participants consented to an additional focus group/interview held in a secure accessible location or via telephone.

**Results:**

Most participants (78.7%) acknowledged the benefits of advance care planning conversations, however, two thirds did not want to think about advance care planning or find out more at present. Respondents were reluctant to broach advance care planning as it was linked to end of life care and funeral plans, and they did not wish to cause distress to their loved one. Respondents trusted their family to respect their wishes and they considered having an advance care plan in place would be of assistance in the future. Top-down leadership, normalisation, and increased education were identified as potential approaches to overcome barriers.

**Conclusions:**

Advance care planning was recognised as important despite limited awareness, lack of knowledge and misperceptions. Whilst a community action approach to enhance understanding and engagement was supported, a ‘one size fits all’ approach will not work; rather bespoke targeting is required with educational and media messaging aligned.

**What is already known about the topic?**

- The ethos of advance care planning should be underpinned by a community engagement approach.
- Internationally, public engagement in advance care planning remains low, owing to lack of awareness and views of irrelevance prior to a health crisis.
- There remains a lack of research on public understanding and attitudes towards advance care planning among the general population.

**What this paper adds?**

- Less than a third of participants (28.5%) had heard the term ‘advance care planning’ and only 7% had engaged in advance care planning.
- Qualitative interviews revealed that the public are reluctant to broach advance care planning as it was linked to the death and dying phase which was considered a social taboo to discuss, and they did not wish to cause distress to their loved one by raising a depressing subject.
- Respondents trusted their family members to respect their wishes however they believed having an advance care plan in place would be of assistance in the future.

**Implications for practice, theory or policy**

-  By shifting the view of advance care planning as a ‘normal’ conversation to have irrespective of health status, essential conversations can start earlier and ensure appropriate care reaches everyone who would benefit.
- Future interventions should consider the role of the family in increasing advance care planning discussions.
- Stereotypical attitudes of advance care planning need to be challenged by offering bespoke communication for different generations with educational and media messaging aligned.
- A consistent message from a trustworthy source, inclusive of the voice of the patient, carer and healthcare professional, and offering both general and tailored information to the needs of specific groups is advocated.

## Background

SARS-CoV-2 (COVID-19) has placed a heightened focus on the importance of advance care planning worldwide, in the context of how health can suddenly deteriorate.^1–3^ Advance care planning is a process that enables adults at any stage or health status to understand, identify and share their personal goals, values and preferences regarding future medical care.^4,5^ It involves ongoing discussions between patients, proxy decision makers and healthcare providers. In the UK, advance care plans can be formal (e.g. advance decisions or assigning a lasting power of attorney) or informal (advance statements regarding what the person considers important to their health and care).^6^ Advocated in policy,^3,7,8^ the overall goal is to ensure congruence between a person’s values and the care received. Despite being recognised by patients and families as a difficult topic for discussion,^9–11^ emerging evidence pre and during COVID-19 suggests that the public might be willing to engage in advance care planning conversations.^12,13^ It helps to ensure the medical care delivered is consistent to the person’s wishes/values and preferences, reduces decision-making burden and helps families prepare for and cope with bereavement.^14^

Research on advance care planning has continually focused on older patients or in the context of a medical crisis,^10,15,16^ and evidence suggests uptake is low.^17–19^ However, there is increasing recognition that advance care planning should engage the public outside of a medical setting.^20,21^ A scoping review of advance care planning with the public from 2011 to 2020 revealed that while 80%–90% of participants had heard the term, only 10%–41% had a named proxy or written document.^22^ Similar levels of engagement with the public have been reported across the United States,^23^ Europe^24,25^ and the United Kingdom.^26,27^ For example, more than 90% of Americans believed it was important to talk about their wishes with a loved one however less than 30% had initiated this conversation, believing it was not the right time and not something they felt they needed to worry about at that stage.^23^

In an attempt to normalise the conversation and increase public engagement, several social media forums^5^ and community promotion initiatives have been launched.^28,29^ Yet evidence^18^ suggests comfort levels around these discussions depend on one’s personal experience with death and dying as well as their knowledge of advance care planning. There remains a lack of research on understanding and attitudes towards advance care planning among the general population. The aim of the study was to examine public awareness, knowledge and attitudes towards advance care planning and identify strategies to raise awareness within a public health framework.

## Methods

### Design

**Figure 1.**
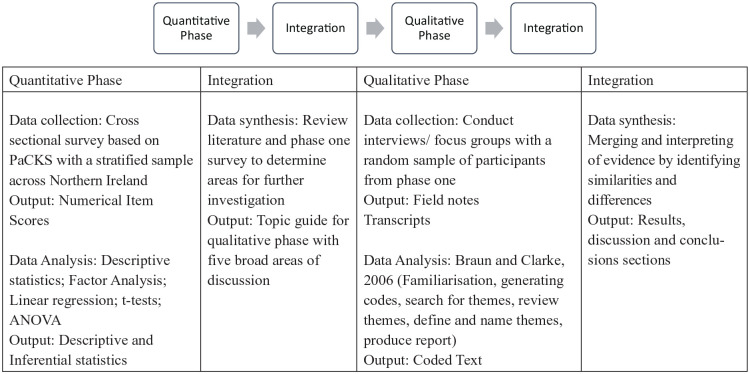
Research design.

A two phase, explanatory, sequential mixed-methods design^30^ was used, including a cross-sectional survey and focus groups/interviews with members of the public. Phase 1 assessed the knowledge, attitudes and behaviours of the public in Northern Ireland regarding advance care planning, and phase 2 explored these concepts in depth and identified future strategies for public promotion of advance care planning. GRAMMS reporting guidelines were used to structure the paper (Figure 1).^31^

### Participants

Participants for the survey comprised a random representative sample of adults from the Northern Ireland population aged 18 years and over; selected from a database of addresses, where interviewers selected one adult at random for face to face completion of the survey at each address using the ‘next birthday’ method; Consent was obtained prior to completion. Following completion of the survey, participants were asked if they would like to participate in the second, qualitative phase. Those who agreed to contribute had their contact information collated (separately to the survey responses). Willing participants were contacted via telephone. Participants who met the eligibility criteria (Table 1) were sent study information and upon receipt of the consent form, were invited to take part.

**Table 1.** Eligibility criteria (phase 2).

Inclusion criteria	Exclusion criteria
Age 18–80	Experienced bereavement within the last 6 months
Able to speak and read English	
Previously completed NILT survey	
Willing to participate and provide informed consent	

### Data collection

Phase 1 data was collected in 2018, as part of the annual, cross-sectional attitudinal, Northern Ireland Life and Times survey (NILT) (https://www.ark.ac.uk/nilt/). Eight items relating to participants’ attitudes towards advance care planning were informed by the EAPC White Paper on advance care planning^32^ and were responded to using a five-point Likert scale. There were a further two yes/no response items on knowledge of advance care planning, as well as a question allowing participants to detail with whom they had previously discussed advance care planning (nine options). Participant’s sociodemographic characteristics (age, gender, religion, education level, marital status and income) were collected. Face-to-face interviews were carried out using computer assisted personal interviewing, and there was a further self-completion questionnaire which respondents were asked to complete on a tablet, or on paper.

Participants in phase 1 were provided with the following definition of advance care planning:

*‘Advance care planning enables people to define goals and preferences for future medical treatment and care, to discuss these goals and preferences with family and health care providers, and to record and review these preferences if appropriate.’*

In phase 2, data was collected from October 2018 to July 2019 by DM, KC, EB and SMcC (postdoctoral researchers and/or specialist practitioners). The researcher was not known to participants prior to the data collection. The interview schedule was based on the literature and quantitative results, comprising of five broad topic areas surround advance care planning: knowledge; behaviour to seek knowledge and information; perceived accessibility of services and future strategies for promoting public awareness. While focus groups were offered, the majority of participants wanted to undertake interviews, lasting 40–60 min. Data were collected in a neutral public place, the participant’s home or via telephone, and were audio recorded and field notes taken. The data collection tools were piloted with academics and NILT data collectors prior to implementation.

### Data analysis

In phase 1, descriptive statistics to summarise demographic factors and the public’s knowledge and attitudes towards advance care planning were calculated using IBM SPSS v25.0.

Phase 2 data were stored and managed through NVivo 10 Software. Focus groups/interviews were transcribed, anonymised and subject to thematic analysis.^33^ This involved a six-step process of familiarisation, generating codes, searching for themes, reviewing themes, defining and naming themes and producing a report. Themes were derived by exploring patterns, similarities and differences within and across the data in relation to participant’s perspectives on advance care planning. Data analysis was done by two authors (OB and FH) to enhance credibility and trustworthiness.

### Integration

Integration was evident through data transformation between phase 1 and 2,^34^ and merging in the results and discussion.^30^ The data from phase 1 informed the development of the interview schedule utilised in phase 2, and the results from both phases were analysed in parallel, and integrated using data matrix and weaving the thread techniques, and presented thematically throughout the results and discussion.

## Results

### Description of sample

A total of 2161 people were contacted, 1201 of whom completed the survey (response rate 56%), representative of the demographic profile in Northern Ireland. The participants were aged between 18 and 95 years (mean: 61 years). The largest proportion of the population, 17.7%, were aged between 45–54 years. Over half were female (58.3%); most were white (95.5%) and born in Northern Ireland (84.2%). A full demographic profile can be found in Table 2.

**Table 2.** Participants’ demographics (phase 1).

Category	*n* (%)
Gender	
Male	501 (41.7%)
Female	700 (58.3%)
Age	
18–24	87 (7.3)
25–34	174 (14.6%)
35–44	203 (17.1)
45–54	210 (17.7%)
55–64	189 (15.9)
65–74	173 (14.6%)
75–84	121 (10.2%)
85+	31 (2.6%)
Marital status	
Single	378 (31.8%)
Married	514 (43.3%)
Married but separated	57 (4.8%)
Divorced	103 (8.7%)
Widowed	136 (11.4%)
No answer/refused	13 (1.1%)
Description of area lived	
Big city	212 (17.7%)
Suburbs/outskirts of big city	109 (9.1%)
Small city or town	498 (41.5%)
Country village	172 (14.3%)
Farm or home in country	210 (17.5%)
Household income	
Fallen behind prices	591 (49.2%)
Kept up with prices	473 (39.4%)
Gone up by more than prices	52 (4.3%)
Don’t know	85 (7.1%)
Country of birth	
Northern Ireland	1011 (84.2%)
England/Scotland/Wales	72 (6%)
Republic of Ireland	33 (2.7%)
Elsewhere	85 (7.1%)
Ethnic group	
White	1147 (95.5%)
Other	54 (4.5%)
Highest qualification	
Degree or higher	302 (25.3%)
Diploma or equivalent	98 (8.2%)
GCE A Level or equivalent	151 (12.7%)
GCSE (A–C) or equivalent	223 (18.7%)
GCSE (D–G) or equivalent	124 (10.4%)
No qualifications	295 (24.7%)
No answer/refused	8 (0.7%)
Religion	
Catholic	431 (38.2%)
Protestant	496 (43.9%)
No religion	202 (17.9%)
Missing/other religion	72 (6%)

Twenty-five participants contributed to phase 2. Almost all participants (96%) were white; 60% were male and 72% were married or co-habiting. Less than a quarter (24%) were under 50 years, with the largest proportion of participants (36%) aged between 61 and 70 years. Almost half (48%) were retired, and all participants were Christian (Table 3).

**Table 3.** Participants’ demographics (phase 2).

Demographics	*n* (%)	Demographics	*n* (%)
Gender		Ethnic origin	
Male	15 (60%)	White	24 (96%)
Female	10 (40%)	Black African	1 (4%)
Age		Marital status	
30–40	1 (4%)	Married	15 (60%)
41–50	5 (20%)	Separated	2 (8%)
51–60	7 (28%)	Divorced	2 (8%)
61–70	9 (36%)	Cohabiting	3 (12%)
71+	1 (4%)	Single (never married)	1 (4%)
Not answered	2 (8%)	Widow/widower	1 (4%)
		Other	1 (4%)
Employment status		Religious affiliation	
Retired	12 (48%)	Catholic	12 (48%)
Employed	8 (32%)	Protestant	10 (40%)
Unable to work	3 (12%)	Other Christian	3 (12%)
Self employed	2 (8%)		

### Findings from thematic synthesis

Three overarching themes were identified through the merging and integration of qualitative and quantitative data: ‘*Advance care planning is a last resort*’; ‘*It’s inevitable but you don’t talk about it*’ and ‘*If you’re better informed, you’ll make a better decision*’.

### Theme 1: Advance care planning is a ‘last resort’

Nearly one third of survey respondents (28.5%, *n* = 276) had heard of the term ‘Advance care planning’ and yet only 7% (*n* = 66) had ever engaged in a conversation about it. Friends/family (60.6%, *n* = 40), the General Practitioner (GP) (43.9%, *n* = 29) and a member of the clergy (31.8%, *n* = 21) were most likely approached to discuss the topic by those who reported that they had engaged in conversations, and in many cases multiple people were approached. Further qualitative exploration found the participants were unaware of the term advance care planning, including those with caregiving experience. Many admitted they were unsure of how best to plan or support someone through a terminal illness with most viewing advance care planning as a ‘last resort’ when all treatment had failed. Perceived to be an ‘insurance’, some participants described it as a ‘legal document’, ‘will’ or ‘contingency plan’ intended to act as a buffer against life events relating to their health. Phase 2 participants noted advance care planning was focused on funeral wishes and medical care/treatment options once a terminal illness had been diagnosed, however, this topic was not considered part of a normal conversation.

Several issues were outlined including a perceived lack of knowledge and awareness by healthcare professionals (HCPs) of advance care planning; and a lack of funding and resources within the health service to facilitate the growing need to promote advance care planning:

*‘. . .putting in place, a plan for what sort of care you might need, different circumstances, different health issues. . . for a service to be available when needed and be locally accessible and available quickly. . .’* (PCACPI010)

For the few participants who knew about advance care planning, information was sourced from online platforms, the media, friends and family, HCPs and religious and social service providers. All participants would approach a healthcare professional (GPs, a consultant if under the care of one, social worker and specialist palliative care teams) if they needed more information about advance care planning. ‘*Asking google*’ was another source of information, however, participants sometimes worried about the credibility of online information due to media reports of false news.

### Theme 2: ‘It’s inevitable, but you don’t talk about it’

Almost two thirds (63.3%, *n* = 578) of respondents felt they were in good health and did not want to think about advance care planning (Table 4). However, when exploring the impact and role of the family, 81.2% (*n* = 732) of respondents felt it would be comforting to know they had left wishes with their family and 66.6% (*n* = 597) felt their wishes would be followed. Respondents believed advance care planning would not have a negative impact on the quality of care they received and 83.6% (*n* = 751) trusted their family to make the right decision for their care. All survey respondents were asked if they would like to find out more about advance care planning and almost two thirds who responded (68.3%, *n* = 607) said no.

**Table 4.** Participants’ responses to items on their attitudes to advance care planning.

Statements	SA	*A*	*N*	*D*	SD	DK
1. I am in good health and do not want to think about preparing an advance care plan	25.4% (232)	37.9% (346)	15.6% (142)	16.3% (149)	3.4% (31)	1.3% (12)
2. It would comfort me to know I have left guidance about my wishes for my family (P)	27.1% (244)	54.1% (488)	10.8% (97)	4.4% (40)	0.4% (4)	3.2% (29)
3. I would worry I could not change my mind (N)	2.2% (20)	16.7% (150)	17.1% (1545)	46.9% (421)	11.1% (100)	5.9% (53)
4. I trust my family to make the right decisions for me (P)	36.4% (327)	47.2% (424)	9.8% (88)	3.7% (33)	1.0% (9)	1.9% (17)
5. I cannot change what will happen in the future and so there is no point in planning (P)	4.0% (36)	18.3% (164)	21.1% (189)	42.7% (383)	11.0% (99)	2.9% (26)
6. It is difficult to know if my wishes will be respected (N)	2.7% (24)	13.5% (121)	14.5% (130)	50.2% (450)	16.4% (147)	2.8% (25)
7. I worry that if I make plans for my future care and treatment, doctors would stop treatment too soon (N)	1.8% (16)	11.9% (107)	17.0% (152)	48.9% (438)	12.5% (112)	7.9% (71)
8. Discussing my wishes would give me a sense of control (P)	23.7% (212)	55.0% (493)	11.4% (102)	5.9% (53)	0.6% (5)	3.5% (31)

Whilst some had started to think about future care, most found the subject difficult to broach with loved ones or HCPs. This was because they feared causing ‘*upset or distress*’ to their loved ones by speaking about ‘*their own mortality*’.

*‘. . .it’s never really talked about . . .like deaths and funerals – nobody really likes to envisage the end. . . it’s inevitable at some stage, but you don’t talk about it, it’s not going to happen, so to speak’.* (PCACPI003)

Some acknowledged that introducing the topic of advance care planning among family and friends could be perceived as an indication of an impending problem:

‘*they’d start to think, is there something wrong with you?*’ (PCACPI014);

or viewed as depressing:

‘*you might be short of friends every time you start talking about death.*’ (PCACPI016).

Respondents in phase 2 felt life was ‘*hectic*’ and ‘*busy*’ and reported the stereotypical view that advance care planning was for those who were older or for when a ‘*health crisis is looming*’. Generational differences were also noted, with a belief that older people may be more able to acknowledge their mortality, whilst younger generations felt it wouldn’t ‘*affect them*’. There was a recognition that such attitudes can be challenging, and participants suggested a more tailored educational approach should be offered for different generations.

*‘once you get to my age (67 years), or getting into your later life, you start to think of things like that (*advance care planning*), whereas . . .younger people are not really going to be interested’* (PCACPI008).

Several participants further reported how their cultural beliefs would influence when, how and who they would approach to discuss advance care planning. However, respondents believed that if they didn’t think about their own mortality, ‘*it’s almost superstition, it won’t happen*’.

Participants also acknowledged that having a shared experience (being directly affected) often facilitated discussions:

‘*whenever the people that you know are going through that process, then the terminology is used loosely, because you’re in that circle’* (PCACPFG002).

### Theme 3: ‘If you’re better informed, you’ll make a better decision’

Whilst advance care planning was viewed as an ‘*individual responsibility*’, participants noted the need for government leadership to aid its implementation into everyday life. It was noted that integrating advance care planning into existing events such as applying for life insurance schemes could stimulate family discussions on the subject. Yet, participants recognised a dearth of standardised lay information hampered public engagement. They recommended that information should be tailored to convey positivity such as the concept of a ‘*a good death*’ and dying in a ‘*good way*’, and broached with everyone, irrespective of their age or health status. Participants added that disseminating such information could be done through various platforms such as information in GP surgeries, libraries, posting leaflets and regionally incorporating media platforms (e.g. TV, radio, newspapers and billboards). There was some discussion about the benefit of using social media platforms and weaving the topics into soap storylines, as a way of gearing people’s mind-set towards advance care planning and stimulate discussions that will ultimately sensitise people:

*‘If you’re better informed, you’ll be able to make a better decision’*; whilst another said, *‘it would have to be a wider thing, it’s about getting information and also* [getting] *it in a way that* [people] *understand’*.

Normalising the conversation as part of everyday language was key for several participants. There was a sense that introducing these conversations informally within families and amongst peers (e.g. in church or social groups) was the best way to break down existing barriers and ‘*taboo*’ surrounding death. They suggested including advance care planning information within the school curriculum as a way of normalising these conversations:

‘. . .*people should be taught about advance care planning at school. You’re thinking about advance care planning and it’s part of the curriculum and lessons, so that when your mum or dad come to have something, you’ve got an awareness instead of it just hitting you like a brick wall and when you come to have your care, your children will be able to support you . . .’* (PCACPI002).

## Discussion

### Main findings

Most participants had not heard the term advance care planning, did not participate in discussions, and did not want to find out more about the topic. Almost two thirds (63.3%, *n* = 578) of survey respondents felt they were in good health and did not want to think about advance care planning, mirroring international and national literature regarding awareness^35,36^ and uptake.^23–27^ Of those who had heard the term, common misperceptions persisted: (1) focus only on medical care and treatment options, (2) only applicable at the end of life and (3) only as a ‘last resort’ when all treatment had failed. The ambivalence of participants, and feelings of ‘*not being ready*’ to engage in advance care planning discussions, mirrors the research base.^11^ However, a review of communication about care goals found that absent, delayed or inadequate communication about end-of-life preferences is associated with poor quality of life and anxiety, family distress, prolongation of the dying process, undesired hospitalizations, patient mistrust of the health care system, physician burnout and high costs.^37^ By waiting until a medical emergency arises, the emotive context of such conversation is raised, which may impact on the clinical, legal and professional ethical decisions made.^38^ In addition, advance care planning at this late stage hinders upstreaming and normalisation of the early conversations advocated in public health policy.^8^ Therefore, increasing awareness and understanding, both among the wider public and health and social care professionals, is crucial. Efforts should be made to integrate advance care planning into bigger public health campaigns on healthy ageing as part of everyday conversations, including funding and resource planning.

Misconceptions have been attributed to lack of public education,^5,39^ lack of exposure to the topic and personal experience of family and friends at the end of life discussing resuscitation, treatment and symptom control.^40^ As a result of these misconceptions, advance care planning is viewed medically and associated with end of life care and may explain why the majority of participants did not want to receive more information. The combination of these factors: (1) impact on viewing advance care planning holistically; (2) may result in less engagement as its not perceived to be relevant and (3) results in non-medical aspects of care not being adequately addressed. To address key misperceptions requires agreement on the key components of the message, shared terminology and consistency in delivery.

Qualitative data from this study revealed that although people acknowledged the benefits of advance care planning, they recommended the need to introduce this at an earlier stage through schools and colleges. Doing so may help to educate and dismantle the cultural taboos and superstitions around death and dying as reported in this study and others.^8^ Evidence suggests that young and old are willing to hold such conversations,^41^ but require a platform through which such conversations can occur. In this study, participants identified opportunities to provide thinking space and facilitate conversations outside of an end of life situation. For example, integrating advance care planning into life insurance policy, story lines in television programme and informal sessions in local community groups. These approaches have already shown some successes.^5,20,28,42^ The pandemic may also offer such a platform and space to create acceptable reasons for the public to discuss future care.^13^ Exemplars of good practice illustrating how community support can be developed and maintained should be developed and provided.

A key theme coming throughout the qualitative data was the need to have a trigger in terms of participating but also the role and importance of family members. Whilst 72% of respondents in the survey were married or cohabitating the influence of this on the respondent’s engagement or attitude to advance care was not reported, despite research indicating that family opinions have been found to be highly influential on the willingness to engage in palliative care and advance care planning discussions.^43,44^ The respondents noted that they would trust their family members to respect their wishes and that they considered that having an advance care plan in place would be of assistance in the future. However, many family caregivers perceive the initiation of conversations about death and dying as burdensome, partly because they lack accurate knowledge and partly because they are unsure how much their loved one wanted to be involved or expects them to be involved.^45^ Therefore, further research should consider a focus on the role of the family and provide them with the knowledge and skills to initiate, and support a loved one through, advance care planning discussions.

### Strengths and weaknesses

Utilising a mixed methods approach helped to contribute to a broader understanding of the publics views however this was with a small convenience sample and hence bias may be introduced. In addition, due to ethical considerations given to the qualitative data collection, we excluded people who had been recently bereaved, therefore, this may have skewed results as the recently bereaved may have had a different perspective. Data were collected prior to the outbreak of the pandemic; therefore, it is possible that attitudes may have changed in terms of willingness to seek out more information or discuss advance care planning. Three researchers from different backgrounds and specialties were involved in data collection, and data analysis was completed by two independent researchers and reviewed by a team member separate from the data collection process to ensure rigour. Phase 2 participants had all participated in phase 1 where a definition of ACP was provided. Although there was a time delay between participation in phases 1 and 2, the impact of this definition on their understanding was not explored in depth.

### What this study adds

This study provides empirical evidence on the knowledge and attitudes of one region of the United Kingdom public’s awareness regarding advance care planning and offers strategic direction to increase awareness and engagement through education within a public health campaign. Despite being advocated in policy^3,7,8^ and embedded in initiatives worldwide, the public are still largely unaware of the term ‘advance care planning’ and what it means, and have a reluctance to engage in discussions, associating it with death and the very end of life. Family members play a key role in influencing attitudes and engagement with advance care planning and should be central in future interventions to increase participation.

By shifting the view of advance care planning as a ‘normal’ conversation to have irrespective of health status, essential conversations can start earlier and ensure appropriate care reaches everyone who would benefit. A community action approach to enhance understanding and engagement is supported, however, a ‘one size fits all’ approach will not work; rather bespoke targeting is required with educational and media messaging aligned. There is a need for public health campaigns to recognise the disparity in what palliative care is seen to offer (end of life care), and move forward by raising awareness, removing misconceptions and increasing openness to holistic palliative care. A consistent message from a trustworthy source, inclusive of the voice of the patient, carer and healthcare professional, and offering both general and tailored information to the needs of specific groups is advocated. Although the results from this study indicate that most people were not interested in seeking out further information, a global shift in mindset resulting from COVID-19 may create a platform upon which to start conversations.

## References

[1] Selman L Lapwood S Jones N , et al. What enables or hinders people in the community to make or update advance care plans in the context of Covid-19, and how can those working in health and social care best support this process?, www.cebm.net/oxford-covid-19/ (2020, accessed 4 November 2020).

[2] Curtis JR Kross EK Stapleton RD. The importance of addressing advance care planning and decisions about do-not-resuscitate orders during novel coronavirus 2019 (COVID-19). JAMA 2020; 323(18): 1771–1772. 32219360 10.1001/jama.2020.4894

[3] Care Quality Commission. Joint statement on advance care planning, https://cqc.org.uk/news/stories/joint-statement-advance-care-planning (2020, accessed September 2020).

[4] Sudore RL Lum HD You JJ , et al. Defining advance care planning for adults: a consensus definition from a multidisciplinary Delphi panel. J Pain Symptom Manage 2017; 53(5): 821–832.e1. 10.1016/j.jpainsymman.2016.12.331 PMC5728651 28062339

[5] Biondo PD King S Minhas B , et al. How to increase public participation in advance care planning: findings from a World Café to elicit community group perspectives. BMC Public Health 2019; 19(1): 679. 31159829 10.1186/s12889-019-7034-4 PMC6547442

[6] NICE. Advance care planning and A quick guide for registered managers of care homes and home care services. NICE Communities and Social Care, https://www.nice.org.uk/about/nice-communities/social-care/quick-guides/advance-care-planning (accessed 18 March 2021).

[7] Department of Health. End of Life Care Strategy Promoting high quality care for all adults at the end of life, www.dh.gov.uk/publications (2008, accessed 7 September 2020).

[8] National Palliative and End of Life Care Partnership. Ambitions for Palliative and End of Life Care: a national framework for local action 2015-2020, www.endoflifecareambitions.org.uk (2015, accessed 12 February 2018).

[9] Brinkman-Stoppelenburg A Rietjens JA Van Der Heide A. The effects of advance care planning on end-of-life care: a systematic review. Palliat Med 2014; 28: 1000–1025. 24651708 10.1177/0269216314526272

[10] Weathers E O’Caoimh R Cornally N , et al. Advance care planning: a systematic review of randomised controlled trials conducted with older adults. Maturitas 2016; 91: 101–109. 27451328 10.1016/j.maturitas.2016.06.016

[11] Zwakman M Jabbarian LJ Van Delden J , et al. Advance care planning: a systematic review about experiences of patients with a life-threatening or life-limiting illness. Palliat Med 2018; 32(8): 1305–1321. 29956558 10.1177/0269216318784474 PMC6088519

[12] Funk DC Moss AH Speis A. How COVID-19 changed advance care planning: insights from the West Virginia Center for End-of-Life Care. J Pain Symptom Manage 2020; 60: e5–e9. 10.1016/j.jpainsymman.2020.09.021 PMC7506348 32976940

[13] Hopkins SA Lovick R Polak L , et al. Reassessing advance care planning in the light of covid-19. BMJ 2020; 369: m1927. 32423988 10.1136/bmj.m1927

[14] Detering KM Hancock AD Reade MC , et al. The impact of advance care planning on end of life care in elderly patients: randomised controlled trial. BMJ 2010; 340(7751): 847. 10.1136/bmj.c1345 PMC2844949 20332506

[15] Freytag J Street RL Barnes DE , et al. Empowering older adults to discuss advance care planning during clinical visits: the PREPARE randomized trial. J Am Geriatr Soc 2020; 68(6): 1210–1217. 32157684 10.1111/jgs.16405 PMC7787080

[16] Glaudemans JJ Moll Van Charante E Wind J , et al. Experiences with approaches to advance care planning with older people: a qualitative study among Dutch general practitioners. BMJ Open 2018; 8(11): 24762. 10.1136/bmjopen-2018-024762 PMC6254415 30478126

[17] Ali M Capel M Jones G , et al. The importance of identifying preferred place of death. BMJ Support Palliat Care 2019; 9(1): 84–91. 10.1136/bmjspcare-2015-000878 26408428

[18] Jimenez G Tan WS Virk AK , et al. Overview of systematic reviews of advance care planning: summary of evidence and global lessons. J Pain Symptom Manage 2018; 56(3): 436–459.e25. 29807158 10.1016/j.jpainsymman.2018.05.016

[19] Rocque GB Dionne-Odom JN Sylvia Huang CH , et al. Implementation and impact of patient lay navigator-led advance care planning conversations. J Pain Symptom Manage 2017; 53(4): 682–692. 28062341 10.1016/j.jpainsymman.2016.11.012 PMC6559345

[20] Kellehear A. Advance care planning as a public health issue. In: Rogne L McCune S (eds) Advance care planning: communicating about matters of life and death. New York, NY: Springer, 2013, pp.333–345. https://www.springerpub.com/advance-care-planning-9780826110213.html (accessed 4 November 2020).

[21] Kellehear A. Compassionate communities: end-of-life care as everyone’s responsibility. QJM 2013; 106: 1071–1075. 24082152 10.1093/qjmed/hct200

[22] Grant M Back A Dettmar N. Public perceptions of advance care planning, palliative care, and hospice: a scoping review. J Palliat Med 2021; 24(1): 46–52. 32614634 10.1089/jpm.2020.0111

[23] Institute for Healthcare Improvement. The Conversation Project. Boston, MA, https://theconversationproject.org/ (2018, accessed 9 November 2020).

[24] Osborn R Moulds D Squires D , et al. International survey of older adults finds shortcomings in access, coordination, and patient-centered care. Health Aff 2014; 33(12): 2247–2255. 10.1377/hlthaff.2014.0947 25410260

[25] Daveson BA Bausewein C Murtagh FEM , et al. To be involved or not to be involved: a survey of public preferences for self-involvement in decision-making involving mental capacity (competency) within Europe. Palliat Med 2013; 27: 418–427. 23426845 10.1177/0269216312471883

[26] ComRes. NCPC: dying matters survey, https://comresglobal.com/polls/ncpc-dying-matters-survey/ (2015, accessed 4 November 2020).

[27] Compassion in Dying. Knowledge of end-of-life rights and choices - YouGov poll (2013) and Compassion in Dying. YouGov Poll, https://compassionindying.org.uk/library/knowledge-end-life-rights-choices-yougov-poll-2013/ (2013, accessed 4 November 2020).

[28] Weaver E Vaughan T. Successful intervention: raising awareness of advanced care planning (ACP) in the rural community setting. BMJ Support Palliat Care 2013; 3(2): 233.2–233.

[29] Sinclair C Williams G Knight A , et al. A public health approach to promoting advance care planning to Aboriginal people in regional communities. Aust J Rural Health 2014; 22(1): 23–28. 24460996 10.1111/ajr.12079

[30] Creswell JW Plano Clark VL. Designing and conducting mixed methods research. 2nd ed. Thousand Oaks, CA: Sage Publications, Inc, 2011, 53–106 p.

[31] O’Cathain A Murphy E Nicholl J. The quality of mixed methods studies in health services research. J Health Serv Res Policy 2008; 13(2): 92–98. 10.1258/jhsrp.2007.007074 18416914

[32] Rietjens JAC Sudore RL Connolly M , et al. Definition and recommendations for advance care planning: an international consensus supported by the European Association for Palliative Care. Lancet Oncol 2017; 18: e543– e551. 28884703 10.1016/S1470-2045(17)30582-X

[33] Braun V Clarke V. Using thematic analysis in psychology. Qual Res Psychol 2006; 3(2): 77–101.

[34] Greene JC Caracelli VJ Graham WF. Toward a conceptual framework for mixed-method evaluation designs. Educ Eval Policy Anal 1989; 11(3): 255.

[35] Teixeira AA Hanvey L Tayler C , et al. What do Canadians think of advanced care planning? Findings from an online opinion poll. BMJ Support Palliat Care 2015; 5(1): 40–47. 10.1136/bmjspcare-2013-000473 PMC4345810 24644188

[36] Zhang N Ning XH Zhu ML , et al. Attitudes towards Advance Care Planning and Healthcare Autonomy among Community-Dwelling Older Adults in Beijing, China. Biomed Res Int 2015; 2015: 453932. 26858955 10.1155/2015/453932 PMC4706851

[37] Bernacki RE Block SD. Communication about serious illness care goals: a review and synthesis of best practices. JAMA Intern Med 2014; 174(12): 1994–2003. 25330167 10.1001/jamainternmed.2014.5271

[38] Waller A Sanson-Fisher R Ries N , et al. Increasing advance personal planning: the need for action at the community level. BMC Public Health 2018; 18(1): 606. 29739369 10.1186/s12889-018-5523-5 PMC5941331

[39] Kermel-Schiffman I Werner P. Knowledge regarding advance care planning: a systematic review. Arch Gerontol Geriatr 2017; 73: 133–142. 28802216 10.1016/j.archger.2017.07.012

[40] Schrijvers D Cherny NI. ESMO clinical practice guidelines on palliative care: advanced care planning. Ann Oncol 2014; 25: iii138–iii142. 25210082 10.1093/annonc/mdu241

[41] Mallon A. Examining young people’s knowledge, attitudes and perceptions of palliative care within a public health context. PhD Thesis, Ulster University. https://pure.ulster.ac.uk/en/studentTheses/examining-young-peoples-knowledge-attitudes-and-perceptions-of-pa (2020, accessed 4 November 2020).

[42] Sloan HD Peters T Johnson KS , et al. Church-based health promotion focused on advance care planning and end-of-life care at Black Baptist Churches: a cross-sectional survey. J Palliat Med 2016; 19(2): 190–194. 26840855 10.1089/jpm.2015.0319

[43] Lin CP Evans CJ Koffman J , et al. What influences patients’ decisions regarding palliative care in advance care planning discussions? Perspectives from a qualitative study conducted with advanced cancer patients, families and healthcare professionals. Palliat Med 2019; 33(10): 1299–1309. 31368854 10.1177/0269216319866641

[44] Levoy K Buck H Behar-Zusman V. The impact of varying levels of advance care planning engagement on perceptions of the end-of-life experience among caregivers of deceased patients with cancer. Am J Hosp Palliat Care 2020; 37(12): 1045–1052. 32281390 10.1177/1049909120917899 PMC7484284

[45] Van Eechoud IJ Piers RD Van Camp S , et al. Perspectives of family members on planning end-of-life care for terminally ill and frail older people. J Pain Symptom Manage 2014; 47(5): 876–886. 24035067 10.1016/j.jpainsymman.2013.06.007

